# Impact of complement C3 levels on the development of healthcare-associated infections in intensive care patients: a retrospective case-control study

**DOI:** 10.1080/07853890.2025.2487631

**Published:** 2025-04-07

**Authors:** Chenjuan Wang, Binhao Chen, Zhekai Ying, Mengyuan Shen, Yiling Luo, Tianchen Lin, Dandan Feng, Dongdong Yang, Zhongheng Zhang, Jiannong Wu

**Affiliations:** ^a^ The First Affiliated Hospital of Zhejiang Chinese Medical University (Zhejiang Provincial Hospital of Chinese Medicine), Hangzhou, China; ^b^ Department of Emergency Medicine, Sir Run Run Shaw Hospital, Zhejiang University School of Medicine, Hangzhou, China

**Keywords:** Healthcare associated infections, intensive care, complement C3, immune markers

## Abstract

**Purpose:**

The immune system serves as a critical line of defence against pathogenic microorganisms. To investigate the impact of immune markers, measured within the first 48 h of intensive care unit (ICU) admission, on the incidence of healthcare-associated infections (HAIs) in ICU patients.

**Methods:**

This case-control study included 364 patients admitted from 1 January 2020 to 30 November 2023, receiving immune marker testing within 48 h of ICU admission. Cox proportional hazard models and propensity score matching evaluated immune markers’ association with HAIs risk. Log-rank tests compared time-to-event by C3 levels. All data processing and analysis were performed using R version 4.2.0 (R Foundation for Statistical Computing, Vienna, Austria) and Python version 3.11 (Python Software Foundation, Wilmington, DE).

**Results:**

In total, 258 patients without HAIs (mean [SD] age, 67.24 [17.79] years) and 106 patients with HAIs (mean [SD] age, 73.80 [14.93] years) were included in the final analysis. The HAIs group had older age, longer hospital stay, lower Sequential Organ Failure Assessment (SOFA) scores, and a higher rate of comorbid infections than the non-HAIs group. Also, the HAIs group had a higher proportion of basophils, lymphocytes, monocytes and T suppressor cells (CD3 + CD8+), while the proportion of neutrophils and B cells (CD19+) was lower. After Cox regression analysis and propensity score adjustment, we found that C3 complement levels (HR: 0.40; 95%CI, 0.16–0.98; *p* = .044) influenced the incidence of HAIs. Patients were then divided into high C3 and low C3 groups based on a cut-off value of 0.455 for C3. A time-to-event plot showed that the median time to HAIs occurrence was nine days in the high C3 group and six days in the low C3 group (*p* = .048).

**Conclusions:**

Elevated complement C3 levels may associat with a reduced incidence of HAIs in ICU patients.

## Introduction

Healthcare-associated infections (HAIs), also known as nosocomial infections, are acquired in the hospital and first manifest 48 h or more after admission or within 30 days after discharge [1,2]. HAIs are occurring at an alarming rate. According to the Centers for Disease Control and Prevention, approximately one in 31 hospitalized patients in the United States is likely to develop HAIs every day [3]. Moreover, the World Health Organization’s Global Report on Infection Prevention and Control [4] revealed that more than 24% of annual healthcare-related sepsis cases tend to be fatal, reaching 52.3% in intensive care settings. Thus, early identification and prevention of patients at risk of developing HAIs are of significant importance in reducing their occurrence.

The management of HAIs follows standard goal-directed therapy, which is mainly based on antibiotics and antiviral therapy [5,6]. During infections, neutrophils can be rapidly mobilized from bone marrow to infection sites, where they combat pathogens through enhanced cytotoxicity and extended lifespan. Adaptive immune components including NK cells and T cells can be activated by innate immune cells like dendritic cells (DCs) and macrophages, thereby enhancing defence against viral, parasitic and bacterial pathogens. The complement system, particularly complement C3, is a critical component of the innate immune response, protecting the host against viruses [7], fungi [8] and parasites [9]. However, ICU patients often have severely compromised immune function, increasing their susceptibility to HAIs and other adverse outcomes [10–12]. Beyond the use of antibiotics and intensive care management, immunotherapies show potential. But there is currently a lack of approved effective treatments [12,13]. Identifying patients at risk of HAIs through immune markers and prioritizing their care is of utmost importance.

Intensive care units (ICUs) are the hospital wards with the highest prevalence of HAIs. Patients with normal immune function are less likely to develop serious infections, while those with compromised immune function are susceptible to deadly infections [14,15]. So, in order to clarify the role of immune system defences against HAIs and to find biomarkers that may be critical in this process. We designed a retrospective case-control study to identify immune molecules that influence the occurrence of HAIs in ICU patients.

## Methods

### Selection of participants

Patients were admitted to the First Affiliated Hospital of Zhejiang Chinese Medical University between 1 January 2020 and 30 November 2023 in this retrospective study. HAIs were defined as infections acquired by hospitalized patients during their stay, i.e. infections occurring more than 48 h after admission [16]. Therefore, we chose the same 48-hour time point for the monitoring of immunization indicators.

Inclusion criteria were: (1) age ≧18 years; (2) all of immune markers collected within 48 h of admission;

Exclusion criteria were: those with missing clinical data.

EC of the First Affiliated Hospital of Zhejiang Chinese Medical University approved this study (Approval Number: 2024-KLS-272-01). In accordance with the Commentary on Guideline 10 of the International Ethical Guidelines for Health-related Research Involving Humans [17], the Ethics Committee waived the requirement for informed consent. This decision was based on the fact that researchers obtained data from the hospital’s information department that did not contain personal identifiers, and that the research had minimal impact on the patients involved. All procedures were performed in accordance with the ethical standards laid down in the 1964 Declaration of Helsinki and its later amendments.

### Data collection

For all patients meeting the above criteria, we extracted data on demographics (age, gender), epidemiology, comorbidities, admission and discharge diagnoses, ICU length of stay, total hospital length of stay, laboratory tests, Sequential Organ Failure Assessment (SOFA) scores, Acute Physiology, and Chronic Health Evaluation II (APACHE II) scores calculated within the first 24 h and all outcomes before hospital discharge. According to the hospital infection monitoring standards issued by the National Health Commission of the People’s Republic of China, reporting HAIs is mandatory.

The immune markers included: neutrophil %, eosinophil %, basophil %, lymphocyte %, monocyte %, immunoglobulin A (IgA) concentration (g/L), IgG concentration (g/L), IgM concentration (g/L), complement component 3 (C3) concentration (g/L), complement component 4 (C4) concentration (g/L), white blood cell (WBC) count, B-cells (CD19+) %, CD14+ %, CD4 + CD25+%, HLA-DR+CD14+, Natural Killer (NK) cells %, regulatory T-cells (Tregs) %, Helper T-cells (CD3 + CD4+)%, helper/suppressor T-cell ratio, total T-cells (CD3 + CD45+) % and suppressor/cytotoxic T-cells (CD3 + CD8+) %.

To minimize the potential confounding effects, clinical data on autoimmune diseases [18,19] – such as systemic lupus erythematosus, rheumatoid arthritis and Henoch–Schönlein purpura – were collected for all enrolled patients.

### Statistical analysis

Data processing and statistical analysis were conducted using R version 4.2.0 (R Foundation for Statistical Computing, Vienna, Austria) and Python version 3.11 (Python Software Foundation, Wilmington, DE). The graphs were created using Prism v9.5 (GraphPad, La Jolla, CA). Baseline characteristics for continuous variables conforming to a normal distribution were expressed as mean ± SD, while categorical variables were expressed as numbers (percentage). Analysis of variance, Chi-square test and non-parametric rank sum test were used for intergroup comparisons.

Cox proportional hazards regression model analysis was performed. Univariate Cox regression was initially conducted, and variables with *p* < .1 were selected for multivariate Cox regression. Variables with *p* < .1 after univariate Cox regression were selected for multivariate Cox regression analysis without using stepwise regression. Hazard ratios (HRs) and 95% confidence intervals (CIs) were used to estimate the effect of different admission indicators within 48 h on infection outcomes.

Propensity score matching (PSM) was concurrently employed to control for the effects of confounding factors. PSM was used to adjust for baseline characteristics of patients, using the nearest 1:2 matching with a calliper value set at 0.2.

Receiver operating characteristic (ROC) curve analysis was performed to determine the optimal cutoff value of complement C3 for predicting HAIs. The Youden index (*J* = sensitivity + specificity − 1) was used to identify the threshold that maximizes diagnostic accuracy. The area under the curve (AUC) with 95%CI was calculated to evaluate the discriminative ability of complement C3. Time-to-event analysis was performed to compare the incidence of HAIs between the high C3 and low C3 groups. Kaplan–Meier’s survival curves were constructed to illustrate the cumulative infection rates over time, with patients censored at the time of discharge or loss to follow-up. The log-rank test was used to assess the statistical significance of differences between the two groups, and median time to HAIs with 95%CIs was calculated for each group.

All statistical tests were two-sided, with a *p* value ≤.05, indicating statistical significance.

## Results

### Clinical features of patients

**Figure 1.**
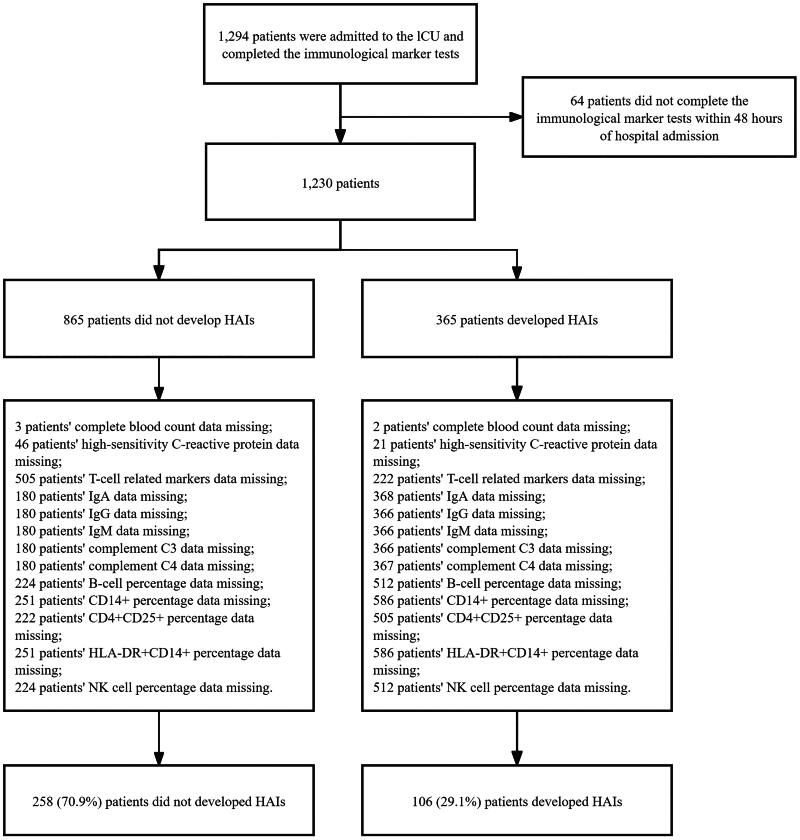
Study flowchart.

During the four-year study period, 1294 patients underwent immune indicator testing during their ICU stay. After excluding 930 patients whose immune markers were not measured within 48 h of admission and those with missing data, 364 patients were included in the final analysis. Among those, 106 (29.1%) patients developed HAIs during hospitalization (Figure 1). Patients in the non-HAIs group were younger, with shorter hospital stay, and higher SOFA scores than the HAIs group (Table 1). The two groups had no significant difference in the APACHE II score (all *p* > .05). A list of possible immune-related diseases affecting patients was provided; there were no differences between groups in trauma, tumours and other diseases (all *p* > .05). There were 85 patients (80.2%) in the HAIs group admitted due to concurrent infection vs. 168 patients (65.1%) in the non-HAIs group.

**Table 1.** Baseline characteristics of enrolled patients.

Variable	Non-healthcare associated infection group (*n* = 258)	Healthcare-associated infection group (*n* = 106)	Total (*n* = 364)	*p* Value
Age (years)	67.24 ± 17.79	73.80 ± 14.93	71.72 ± 15.92	<.001*
Gender, *n* (%)				.481
Male (%)	166 (64.3%)	73 (68.9%)	239 (65.7%)	
Female (%)	92 (35.7%)	33 (31.1%)	125 (34.3%)	
Duration in hospital (days)	15.00 (8.00, 25.00)	39.50 (20.00, 124.25)	18.00 (10.00, 35.00)	<.001*
SOFA	9.00 (5.00, 12.00)	5.00 (4.00, 7.00)	7.00 (4.00, 11.00)	<.001*
Apache II	17.00 (12.25, 23.75)	19.00 (15.00, 22.00)	18.00 (13.00, 23.00)	.158
Complications				
*Infections*, *N* (%)				.007*
Yes	168 (65.1%)	85 (80.2%)	253 (69.5%)	
No	90 (34.9%)	21 (19.8%)	111 (30.5%)	
*Injures*, *N* (%)				.155
Yes	22 (8.5%)	15 (14.2%)	37 (10.2%)	
No	236 (91.5%)	91 (85.8%)	327 (89.8%)	
*Emergency surgery*, *N* (%)				.676^a^
Yes	5 (1.9%)	1 (0.9%)	6 (1.6%)	
No	253 (98.1%)	105 (99.1%)	258(98.4%)	
*Tumour*, *N* (%)				.540
Yes	45 (17.4%)	15 (14.2%)	60 (16.5%)	
No	215 (82.6%)	91 (85.8%)	304 (83.5%)	
*Autoimmune diseases*, *N* (%)				.035*
Yes	44 (17.1%)	9 (8.5%)	53 (14.6%)	
No	214 (82.9%)	97 (91.5%)	311 (85.4%)	
Outcome, *N* (%)				<.001*
Cure	17 (6.59%)	0 (0.00%)	17 (4.67%)	
Recovery	158 (61.24%)	47 (44.34%)	205 (56.32%)	
Not recovered	35 (13.57%)	26 (24.53%)	61 (16.76%)	
Death	39 (15.12%)	26 (24.53%)	65 (17.86%)	
Others	9 (3.49%)	7 (6.60%)	16 (4.40%)	

SOFA: sepsis-related organ failure assessment. Infections, were deemed as infections present at the time of hospital admission, including community-acquired infections, as documented in the medical records.

For non-normally distributed continuous variables, the median (interquartile range) [*M* (P25, P75)] was used, and differences was tested using the nonparametric rank-sum test. Descriptive statistics are presented as mean ± standard deviation (SD) for normally distributed continuous variables, and differences are tested using the *t*-test. Categorical variables were described using counts (%) and analysed using the Chi-square test.

^a^ Fisher’s exact test when performing the Chi-square test.

* *p* < .05 indicating statistical significance.

### Baseline immunological markers

Table 2 shows the differences in immunological markers between the two groups. Patients in the HAIs group had significantly higher proportions of basophils, lymphocytes, monocytes and T-suppressor cells (CD3 + CD8+) compared to the non-HAIs group. In contrast, the non-HAIs group had significantly higher percentages of neutrophils and B cells (CD19+) compared to the HAIs group.

**Table 2.** Immunological markers within 48 h of hospital admission.

Variable	Non-healthcare associated infection (*n* = 258)	Healthcare-associated infection (*n* = 106)	Total (*n* = 364)	*p* Value
Neutrophil %	85.85 (80.23, 91.00)	84.20 (72.85, 90.23)	85.20 (78.60, 90.82)	.022*
Eosinophil %	0.30 (0.00, 1.00)	0.55 (0.00, 2.00)	0.30 (0.00, 1.30)	.061
Basophil %	0.20 (0.10, 0.30)	0.30 (0.10, 0.40)	0.20 (0.10, 0.40)	.044*
Lymphocyte %	7.20 (4.20, 11.15)	7.80 (5.10, 14.25)	7.50 (4.40, 12.00)	.046*
Monocyte %	5.30 (3.40, 7.68)	6.55 (3.50, 9.07)	5.60 (3.40, 7.90)	.027*
IgA (g/L)	2.23 (1.54, 3.14)	2.38 (1.61, 3.40)	2.33 (1.55, 3.23)	.220
IgG (g/L)	9.76 (7.42, 12.30)	9.98 (7.74, 13.05)	9.81 (7.58, 12.50)	.421
IgM (g/L)	0.67 (0.46, 0.97)	0.74 (0.51, 0.95)	0.70 (0.49, 0.96)	.286
C3 (g/L)	0.70 (0.53, 0.89)	0.67 (0.52, 0.87)	0.69 (0.53, 0.88)	.587
C4 (g/L)	0.18 (0.13, 0.24)	0.19 (0.14, 0.24)	0.18 (0.13, 0.24)	.760
WBC (×10^9^/L)	9.70 (7.30, 13.78)	9.30 (7.20, 13.38)	9.60 (7.20, 13.70)	.457
B-cell (CD19+)%	14.66 (8.62, 24.44)	10.78 (4.56, 18.65)	13.86 (7.25, 23.48)	.002*
CD14+%	5.00 (3.02, 7.38)	6.25 (3.52, 7.50)	5.40 (3.18, 7.40)	.078
CD4 + CD25+%	2.20 (1.20, 3.49)	2.20 (1.33, 3.40)	2.20 (1.28, 3.46)	.621
HLA-DR+CD14+%	89.60 (73.20, 96.18)	84.55 (66.05, 95.23)	88.35 (71.18, 95.90)	.251
NK cells %	14.94 (8.96, 24.26)	16.20 (8.84, 23.68)	15.09 (8.94, 24.11)	.799
Treg %	7.00 (5.00, 9.50)	7.40 (5.00, 9.92)	7.00 (5.00, 9.60)	.438
Helper T-cell (CD3 + CD4+) %	36.0 ± 13.1	36.3 ± 11.2	36.1 ± 12.1	.853
Helper/suppressor T-cell ratio	1.68 (1.00, 2.57)	1.52 (0.85, 2.44)	1.58 (0.94, 2.54)	.151
Total T-cell (CD3 + CD45+) %	63.42 (55.50, 72.25)	67.48 (56.73, 75.09)	64.73 (55.65, 73.72)	.054
Suppressor/cytotoxic T-cell (CD3 + CD8+)%	21.51 (15.16, 29.95)	25.65 (16.94, 34.66)	22.25 (15.71, 31.46)	.006*

* *p* < .05 indicating statistical significance.

### Cox proportional hazards regression model

Age, SOFA score, occurrence of extrahospital infection, percentage of eosinophils, percentage of basophils, complement C3, HLA-DR+CD14+, percentage of Treg cells, and percentage of T-suppressor cells were included in the multivariable Cox proportional hazards model. Both crude and adjusted models were analysed. Autoimmune diseases was not significant (*p* = .526) in the univariate Cox analysis and was therefore excluded from the model.

In the crude model, higher levels of complement C3 were associated with a lower risk of HAIs (Table 3). After adjusting for age, occurrence of extrahospital infection, and SOFA score, the association between complement C3 and HAIs risk remained (*p* = .015; HR: 0.32; 95%CI [0.13, 0.80]). Data also showed significant differences in SOFA score and extrahospital infection status (*p* < .05), suggesting that the initial SOFA score and extrahospital infection status were confounding factors for HAIs. Specifically, patients with extrahospital infections had a 122% higher risk of HAIs than those without HAIs (HR: 2.22; 95%CI [1.31, 3.77]; *p* = .003).

**Table 3.** Immunological markers within 48 h of hospital admission and HAIs.

Variable	Model 1^a^		Model 2^b^		Model 3^c^	
	HR (95%CI)	*p* Value	HR (95%CI)	*p* Value	HR (95%CI)	*p* Value
Age	–	–	1.01 (1.00–1.03)	.076	1.01 (1.00–1.03)	.106
SOFA	–	–	–	–	0.88 (0.83–0.93)	<.001*
Complications						
*Infections*		–		.023*		.003*
Yes	–		1.82 (1.09–3.05)		2.22 (1.31–3.77)	
No	–		1.00 (reference)		1.00 (Reference)	
Eosinophil	1.08 (1.00–1.18)	.063	1.07 (0.99–1.16)	.099	1.04 (0.95–1.13)	.423
Basophil	1.32 (0.63–2.76)	.459	1.14 (0.54–2.43)	.731	0.95 (0.43–2.10)	.905
C3	0.42 (0.19–0.96)	.040*	0.56 (0.24–1.30)	.178	0.32 (0.13–0.80)	.015*
HLA-DR+CD14+	0.99 (0.99–1.00)	.157	0.99 (0.99–1.00)	.209	0.99 (0.98–1.00)	.125
Treg	1.05 (1.00–1.11)	.061	1.04 (0.98–1.09)	.171	1.02 (0.97–1.08)	.428
Suppressor/cytotoxic T-cell (CD3 + CD8+)	1.02 (1.00–1.03)	.056	1.02 (1.01–1.03)	.034*	1.02 (1.01–1.03)	.019*

* *p* < .05 indicating statistical significance.

^a^ No adjustment.

^b^ Adjust for age and infections.

^c^ Adjust for age, infections and SOFA score.

### Propensity score matching

After PSM, 137 patients (39%) with a median age of 74 years (IQR 48–68) were classified into the HAIs group. Age, SOFA scores and APACHE II scores were balanced between the two groups, and diseases that could affect immunologic results were matched. Additionally, infections and autoimmune diseases were well-balanced between the groups following PSM. The results after PSM are shown in Table 4.

**Table 4.** Patient characteristics in the propensity score-matched population.

Variable	Non-healthcare-associated infection group (*n* = 137)	Healthcare-associated infection group (*n* = 87)	Total (*n* = 224)	*p* Value
Age (years)	74.00 (63.00, 85.00)	74.00 (66.00, 84.00)	74.00 (64.75, 84.25)	.877
SOFA	6.00 (4.00, 10.00)	6.00 (4.00, 9.00)	6.00 (4.00, 9.00)	.787
APACHE II	17.00 (12.00, 22.00)	19.00 (14.00, 22.00)	18.00 (13.00, 22.00)	.124
*Complications*				
Infections				.501
Yes	100 (72.99)	67 (77.01)	167 (74.55)	
No	37 (27.01)	20 (22.99)	57 (25.45)	
Injures, *N* (%)				.457
Yes	13 (9.49)	11 (12.64)	24 (10.71)	
No	124 (90.51)	76 (87.36)	200 (89.29)	
Emergency surgery, *N* (%)				1.000
Yes	1 (0.73)	0 (0.00)	1 (0.45)	
No	136 (99.27)	87 (100.00)	223 (99.55)	
Tumour, *N* (%)				.081
Yes	30 (21.90)	11 (12.64)	41 (18.3)	
No	107 (78.10)	76 (87.36)	183 (81.7)	
Fundamentals of immunology, *N* (%)				.757
Yes	16 (11.68)	9 (10.34)	25 (11.16)	
No	121 (88.32)	78 (89.66)		

Complications infections, infections that are already present in the patient at the time of admission.

### Cox proportional hazards regression analysis following propensity score matching

**Figure 2.**
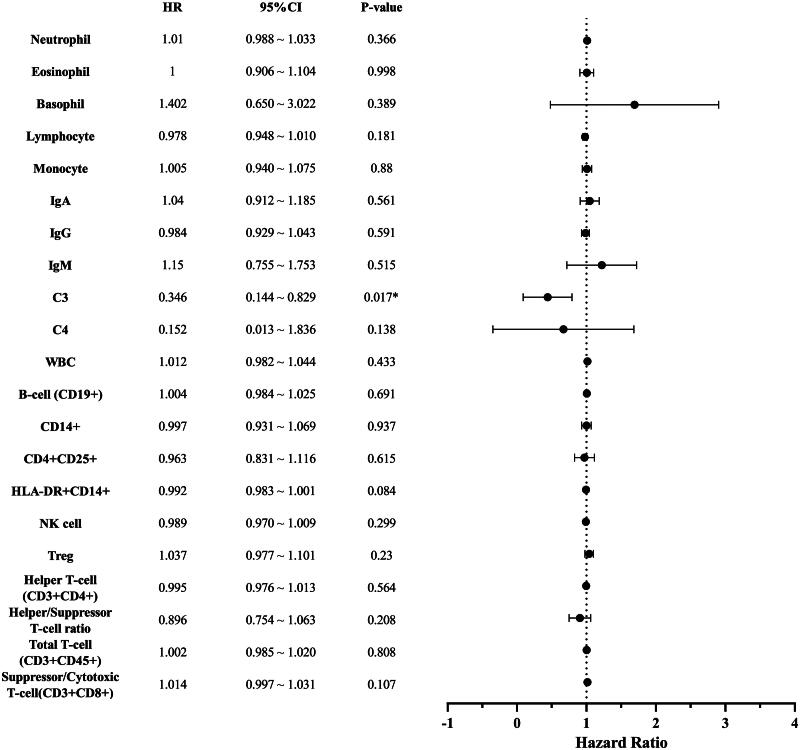
Univariate Cox regression analysis.

**Figure 3.**
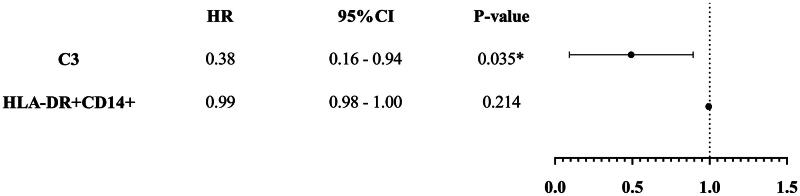
Multivariate Cox regression analysis.

The effect of complement C3 on the incidence of HAIs remained significant in both univariate and multivariate Cox regression analyses (Figures 2 and 3). Each 1 g/L increase in complement C3 was associated with a 60% reduction in the risk of HAIs (HR: 0.40; 95%CI, 0.16–0.98; *p* = .044).

### Cumulative infection rate

**Figure 4.**
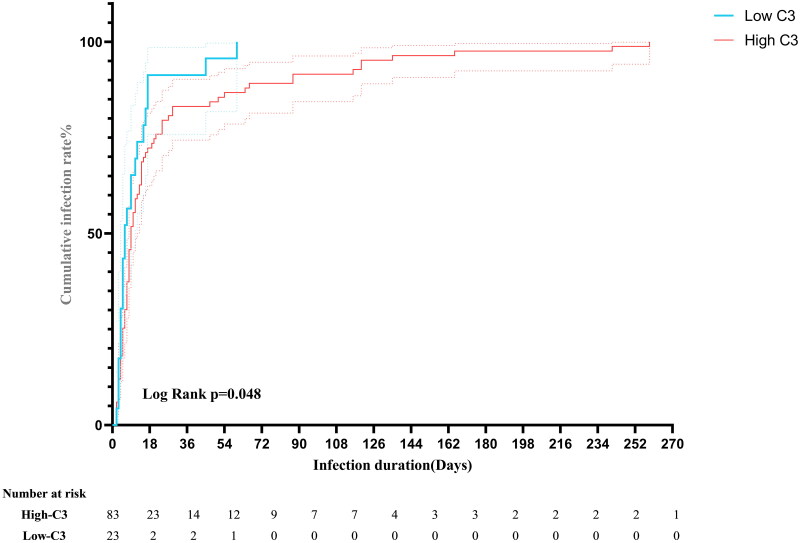
Time-to-event plot.

To explore the potential of complement C3 as a biomarker for infection risk, we analysed its continuous distribution and investigated its association with protective effects against infections. After establishing a new cutoff of C3 = 0.455 using ROC curve analysis (Table S1), the 365 unmatched patients were divided into groups based on their complement C3 levels. *Patients*. A total of 258 patients who did not develop HAIs were excluded from the analysis. Ultimately, 83 patients in the C3 high complement group and 23 patients in the C3 low complement group developed HAIs. A time-to-event plot was created to illustrate the infection times for both groups, as shown in Figure 4. The high C3 group exhibited a longer median time to HAIs (nine days) compared to the low C3 group (six days). The cumulative infection rates between the high C3 group and the low C3 group were compared using Kaplan–Meier’s survival analysis. The log-rank test results showed that the difference in cumulative infection rates between the two groups was statistically significant (*χ*^2^ = 3.94, *p* = .048).

## Discussion

The immune system is a crucial defence mechanism against infections. Previous studies have shown that mHLA-DR may help predict the occurrence of invasive Candida infections in high-risk patients with septic shock. Additionally, a meta-analysis indicated that absolute counts of lymphocyte subsets are associated with the prognosis of COVID-19 patients. To investigate the impact of immune biomarkers on the development of hospital-acquired infections in ICU settings, we analysed a total of 21 immune markers. Our results showed that ICU patients had elevated neutrophil and Treg percentages (as a proportion of CD4+ T cells) and reduced complement C3 and HLA-DR+CD14+ percentages in ICU patients within 48 h of admission, which suggests altered immune functional status in these patients. Similar observations have been reported in the previous studies. For example, changes in HLA-DR expression on monocytes have been found in post-traumatic sepsis patients admitted to ICU [20,21]. Also, increased Treg percentages (as a percentage of CD4+ T cells) early in the course of the disease have been found to indicate a significant increase in Tregs [22]. Furthermore, immune status may be related to infections and influenced by changes in various immunological markers due to trauma, tumours and autoimmune diseases [23–25]. Patients admitted to ICU often present with complex illnesses and may suffer from multiple diseases simultaneously, which may account for the differences in initial immunologic markers observed in this study.

Our results suggested that older patients with previous community-acquired infections were more susceptible to HAIs compared to younger patients, revealing that previous infections may increase the chances of current infections. Similar observations were observed in studies exploring the influence of age on sepsis [26], where both younger and older patients were found to have significant decrease in absolute lymphocyte counts after sepsis. However, while younger patients generally recover to normal lymphocyte counts by day 14, older patients often remain lymphopaenic for up to 28 days after disease onset, with reduced lymphocyte counts being closely associated with severe infections [27,28].

Our study suggested that complement C3 levels may influence the occurrence of HAIs. Currently, there is no established standard for defining high and low levels of complement C3 in relation to the risk of HAIs. Therefore, to investigate the differences between high and low complement C3 levels, we conducted further analyses. The results suggest that higher complement C3 levels may have a protective effect against HAIs in ICU patients. Complement was considered an ‘underappreciated therapeutic target’ [29]. The complement system, a vital component of innate immunity, serves as a link between innate and adaptive immune responses [30,31]. It directly lyses foreign cells and helps clear immune complexes when combined with IgM and IgG to exert antimicrobial effects. Complement C3 is central to the three main extracellular activation pathways [32,33]. The liver is the main organ for its synthesis. It is produced by most cell types [34] and is widely present in the bloodstream, tissues and even within cells, with the liver serving as its primary site of synthesis. This expression contributes to its antimicrobial activity in blood and tissue fluids. In this study, we detected differences in complement C3 levels within 48 h of admission in patients with HAIs. After adjusting for propensity scores, the results did not change. The normal range for complement C3 is 0.79–1.52 g/L; in this study, the mean values were 0.67 g/L in the HAIs group and 0.70 g/L in the non-HAIs group, which is consistent with previous studies showing decreased C3 levels in sepsis patients [35–37]. Additionally, studies on intestinal pathogen infection and immunity in mice have indicated that intestinal complement systems, functioning as sentinels in the human gut, have a crucial role in preventing intestinal infections, with baseline C3 levels being especially significant [38]. However, immunity system can be a double-edged sword, as excessively high or low complement levels can also contribute to disease [39–41], thus highlighting the need for further investigation of the nonlinear relationship between complement C3 levels and prevention of HAIs.

The present study has a few limitations. We used various statistical methods to control for confounding factors affecting the outcome. However, the unmatched Cox regression analysis showed the influence of SOFA scores and the occurrence of out-of-hospital infections on the results, suggesting that these two confounders may directly influence the analysis. Although we controlled for the occurrence of extrahospital infections, the duration and treatment of such infections were not explicitly considered. Despite these limitations, our study highlights the potential importance of complement C3 in critically ill patients. The association between low complement C3 levels and an increased risk of HAIs suggests that monitoring C3 levels could help identify patients at higher risk of HAIs for timely intervention and potentially improve results. Future research should address these limitations by considering the timing and treatment of extra hospital infections and broadening the spectrum of diseases that influence immune status, which could help refine the predictive power of C3 levels and improve understanding of their role in the immune response of critically ill patients.

## Conclusions

ICU patients with relatively higher levels of complement C3 are less likely to develop HAIs. Our data suggests that complement C3 levels detected within the first 48 h in ICU patients may be a key factor influencing the occurrence of HAIs.

## Supplementary Material

Supplementary Table.docx

## Data Availability

The data that support the findings of this study are available from the corresponding author upon reasonable request.
