# Supplementary material for: Impact of complement C3 levels on the development of healthcare-associated infections in intensive care patients: a retrospective case-control study
Source: Ann Med. 2025 Apr 7;57(1):2487631. doi: 10.1080/07853890.2025.2487631 (PMC11980203; doi:10.1080/07853890.2025.2487631)
Supplement: Supplementary Table.docx [file IANN_A_2487631_SM4347.docx]

**Supplementary Table**

**Table S1.** Cut-off Value for Complement C3 with an ROC Curve

| AUC (95%CI) | Accuracy (95%CI) | Sensitivity (95%CI) | Specificity (95%CI) | PPV (95%CI) | NPV (95%CI) | Cut off |
| --- | --- | --- | --- | --- | --- | --- |
|  |  |  |  |  |  |  |
| 0.56 (0.48-0.63) | 0.36 (0.30-0.43) | 0.12 (0.06 - 0.17) | 0.75 (0.66 - 0.84) | 0.42 (0.26 - 0.58) | 0.35 (0.28 - 0.42) | 0.455 |
